# Single-cell transcriptome profiling of buffelgrass (*Cenchrus ciliaris*) eggs unveils apomictic parthenogenesis signatures

**DOI:** 10.1038/s41598-021-89170-y

**Published:** 2021-05-10

**Authors:** Yuji Ke, Maricel Podio, Joann Conner, Peggy Ozias-Akins

**Affiliations:** 1 Institute of Plant Breeding, Genetics and Genomics, University of Georgia, Tifton, GA 31793 USA; 2 Department of Horticulture, University of Georgia, Tifton, GA 31793 USA

**Keywords:** Developmental biology, Genetics

## Abstract

Apomixis, a type of asexual reproduction in angiosperms, results in progenies that are genetically identical to the mother plant. It is a highly desirable trait in agriculture due to its potential to preserve heterosis of F_1_ hybrids through subsequent generations. However, no major crops are apomictic. Deciphering mechanisms underlying apomixis becomes one of the alternatives to engineer self-reproducing capability into major crops. Parthenogenesis, a major component of apomixis, commonly described as the ability to initiate embryo formation from the egg cell without fertilization, also can be valuable in plant breeding for doubled haploid production. A deeper understanding of transcriptional differences between parthenogenetic and sexual or non-parthenogenetic eggs can assist with pathway engineering. By conducting laser capture microdissection-based RNA-seq on sexual and parthenogenetic egg cells on the day of anthesis, a de novo transcriptome for the *Cenchrus ciliaris* egg cells was created, transcriptional profiles that distinguish the parthenogenetic egg from its sexual counterpart were identified, and functional roles for a few transcription factors in promoting natural parthenogenesis were suggested. These transcriptome data expand upon previous gene expression studies and will be a resource for future research on the transcriptome of egg cells in parthenogenetic and sexual genotypes.

## Introduction

Apomixis is commonly defined as a reproductive phenomenon in angiosperms where embryos form from maternal cells in the ovule without meiosis and syngamy, resulting in asexual seed formation^1–3^. Apomixis is widespread in flowering plants and has now been described in 148 genera as adventitious embryony where the embryo arises from somatic cells in the ovule, 110 genera as aposporous and 68 genera as diplosporous where the embryo develops from the unreduced egg in a nucellar or megaspore mother cell-derived embryo sac, respectively^4^. While apomicts are by nature clonal and can produce seeds of identical genotype to the mother plant, no major crops have this self-reproducing capability. Elucidating molecular mechanisms of apomixis will significantly enhance the plant breeding toolbox for preserving genetic composition of elite hybrid cultivars.

Significant strides have been made in deciphering the genetic control of apomixis in a wide range of natural apomicts^3^, although genes underlying the multiple components comprising apomixis are still not fully elucidated. In the natural apomict *Pennisetum squamulatum* (syn*. Cenchrus squamulatus*), mapping in a F_1_ population derived from a cross between *Pennisetum glaucum* (syn. *C. americanus*; pearl millet) x *P. squamulatum* segregating for apospory and sexuality revealed 12 apospory-linked markers, which defined a contiguous apospory-specific genomic region (ASGR)^5^. The linkage of many of these markers to apospory was further shown in *C. ciliaris*^6^. The physical size of the ASGR in *P. squamulatum* was determined to encompass ~ 50 Mb based on fluorescent in situ hybridization mapping of multiple ASGR-linked and one ASGR-recombinant BAC clones. The physical size of the ASGR in *C. ciliaris* could not be determined due to a lack of identified ASGR-recombinant BAC clones; however, the co-linearity of shared ASGR-linked BAC clones between the two species suggested that the two apomictic species share a similar physical size^7–9^. Sequencing of ASGR-linked bacterial artificial chromosome clones from *P. squamulatum* and *C. ciliaris* led to the identification of genes within the region including an AP2-domain containing transcription factor (*ASGR-BABY BOOM-like, ASGR-BBML*)^10,11^. The *PsASGR-BBML* transgene was shown to induce parthenogenesis in sexual pearl millet as well as in maize and rice, but not in *Arabidopsis*^12,13^.

Comparative genomics and transcriptomics studies of apomictic plants and their sexual relatives or siblings often can help unlock the functional molecular components of apomixis that have not been genetically tractable. However, genomes of most natural apomicts remain unsequenced, and targeting the critical tissues where components of apomixis are expressed for RNA-seq has been biologically and technically challenging^14^. Recent expression studies have focused on and advanced our understanding of the first component in the apomixis pathway, termed apomeiosis, by investigating differentially expressed molecular signatures between sexual and apomictic reproductive tissues. Apomeiosis encompasses the initiation of mitotic divisions leading to the production of unreduced embryo sacs derived either from a somatic cell of the ovule nucellus (apospory) or from a megaspore mother cell (diplospory). Differential expression in whole ovules, each containing one to a few apomeiotic cells, to more targeted cells isolated using laser capture microdissection (LCM) has been investigated^15–17,21–23^. Gene expression activity within monocots prior to and after fertilization has been interrogated in isolated egg/zygote cells of maize and rice, with an emphasis on zygotic genome activation^24,25^. An important missing component in current expression studies is the transcriptomic comparison between sexual and parthenogenetic egg cells. Parthenogenesis, embryo development of the egg cell without fertilization, is the second component of the apomixis pathway, with transcriptomic analysis providing an approach to expose pathways underlying natural parthenogenesis.

Discovery of genes driving parthenogenesis in the natural apomict *C. ciliaris* has been challenging due to limited genomic resources and technical difficulties in accessing the egg cell expressing parthenogenesis. Droplet-based single cell RNA-seq recently has been applied to acquire gene expression profiles for complex tissues such as *Arabidopsis* roots and developing ears of maize^26,27^. Yet it remains challenging for the current technology to capture extremely rare transcripts underlying core traits of interest in sparse and spatially-restricted cells like eggs. By conducting laser capture microdissection (LCM)-based RNA-seq on unfertilized sexual and parthenogenetic eggs on the day of anthesis and deep sequencing, combined with de novo transcriptome assembly and computational analyses, we created a de novo transcriptome with sequence information for *C. ciliaris* eggs. Transcriptional profiles that distinguish the parthenogenetic egg from its sexual counterpart were identified, and suggested functional roles for a few key transcription factors and pathways in promoting natural parthenogenesis. Our transcriptome data complement previous gene expression studies and will be an important resource for research on natural parthenogenesis.

## Results and discussion

### Parthenogenetic embryo frequency

Twelve percent of unpollinated ovaries from obligately apomictic genotype B-12–9, two days after anthesis, were shown to contain parthenogenetic embryos (Supplementary Fig. S1; Table 1) as evidenced by the presence of a multicellular embryo without endosperm development deduced from retention of the polar nuclei. This frequency of parthenogenesis in unpollinated ovaries of apomictic *C. ciliaris* is consistent with observations of Veille-Calzada^28^ who reported 7–27% proembryos in unpollinated ovaries of different genotypes. No parthenogenetic embryos were observed in the sexual genotype B-2s. These data confirm the apomictic and sexual phenotypes of the plants used in this experiment and the obligate nature of sexuality for B-2s.

**Table 1:** Parthenogenetic embryo frequency in sexual and apomictic ovaries on Day0 and Day2.

Genotype/date	Number of ovaries observed	Number of ovaries containing parthenogenetic embryos	Frequency of parthenogenetic embryos
B-2s Day0	50	0	0
B-2s Day2	50	0	0
B-12–9 Day0	50	0	0
B-12–9 Day2	50	6	12%

### Egg identification and collection

**Figure 1:**
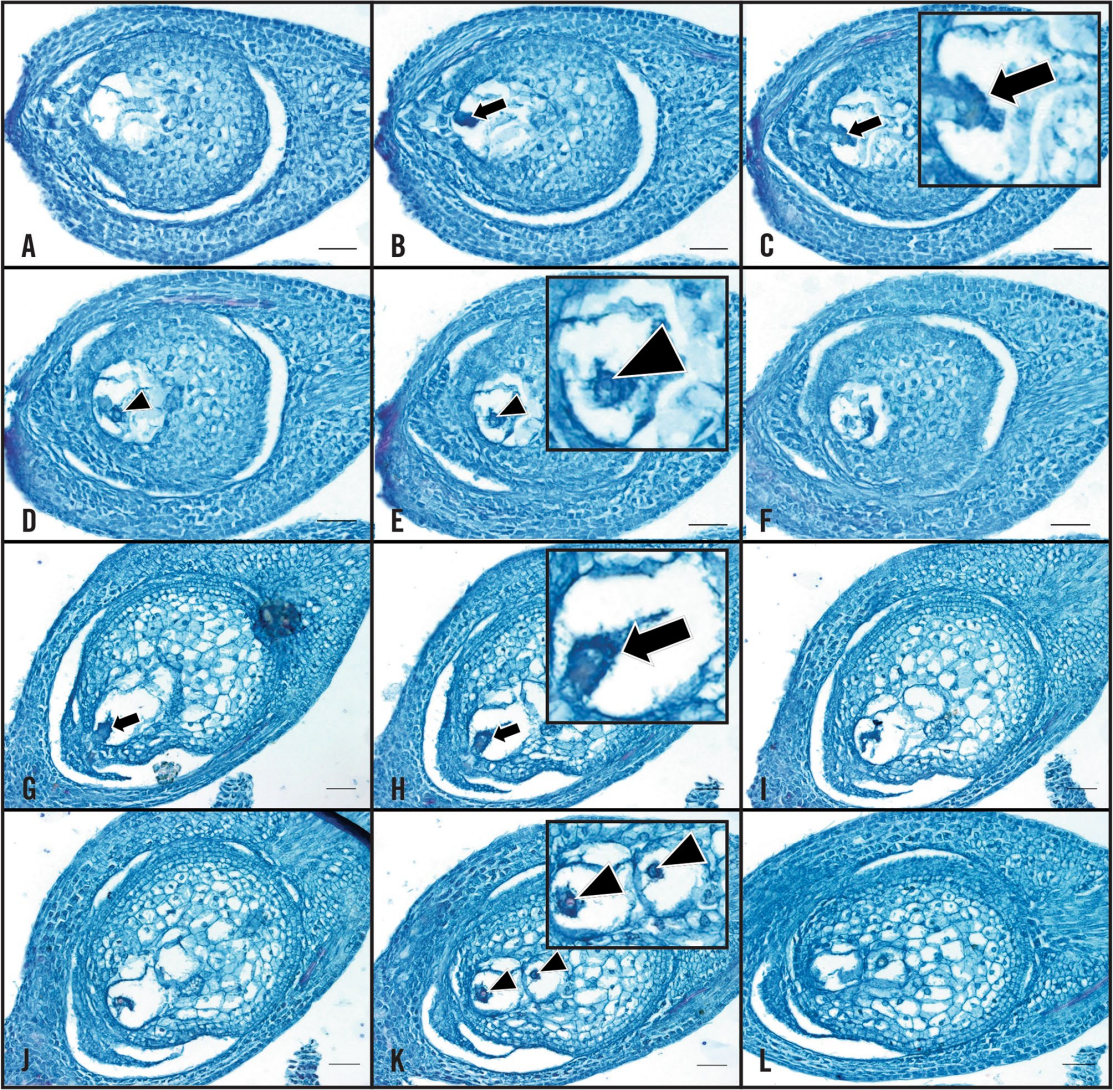
Buffelgrass ovary sections stained with Safranin & FastGreen. (**A**–**F**) Serial sections of sexual genotype B-2s showing a single sexual embryo sac. (**G**–**L**) Serial sections of aposporous genotype B-12–9 showing multiple aposporous embryo sacs. Egg cells are indicated by arrows and polar nuclei are indicated by triangles, scale bar = 50 μm. Enlarged insets have been provided for some images.

Serial sections of sexual *C. ciliaris* genotype B-2s showed a single sexual embryo sac containing the egg apparatus at the micropylar end, two polar nuclei, and antipodals at the chalazal end (Fig. 1A–F). In aposporous genotype B-12–9, an egg apparatus and polar nuclei, but no antipodals, were observed in the aposporous embryo sac nearest to the micropylar end of the ovule (Fig. 1G–L). An additional aposporous embryo sac also was frequently detected. This is consistent with previous descriptions of aposporous embryo sac development in *C. ciliaris*^29^.

### De novo egg, ovule, and ovule plus egg transcriptome assemblies

De novo assembly and annotation of the *C. ciliaris* egg transcriptome was challenging for several reasons. (1) No annotated reference *C. ciliaris* genome sequence is available to guide transcriptome assembly. (2) The ultra-low input quantity of egg cell RNA required multiple cycles of cDNA amplification for downstream library preparation, potentially introducing PCR-generated nucleotide errors within the sequencing reads resulting in an excess of allelic variants. (3) The inevitable compromised quality of LCM-derived RNA and a poly-A based cDNA synthesis strategy resulting in the loss of 5′mRNA ends^30^.

To partially address these challenges, high-quality, abundant ovule RNA was used for amplification-free cDNA library construction. The resulting reads were used to improve the assembly and annotation of LCM egg reads based on the hypothesis that the egg, as a biological subset of ovule tissues, will be represented in the whole ovule transcriptome with more complete transcripts than in the LCM egg transcriptome. Therefore, three de novo assemblies were constructed using Trinity: ovule only, LCM egg, and LCM egg plus ovule. Statistics, metrics for completeness, and number of unique plant annotations for each assembly were compared (Table 2).

**Table 2:** Basic statistics, completeness and number of unique plant hit descriptions for each de novo assembly.

	LCM egg	ovule	LCM egg plus ovule
Total Trinity transcripts	151,167	214,836	285,510
Total trinity ‘genes’^1^:	91,742	87,787	130,601
Contig N50 based on transcripts (bases)	433	1,747	1,527
Contig N50 based on ‘genes’ (bases)	454	1,595	1,163
Overall alignment rate^2^	77.6%	96.1%	92.9%
# of plant hit descriptions (annotation through NCBI nt database)	70,650	137,724	157,523
# of unique plant hit descriptions^3^	23,049	33,826	36,861
% of complete BUSCOs^4^	10.2%	88.6%	85.1%
% of fragmented BUSCOs	17.8%	6.7%	9.3%
% of missing BUSCOs	72.0%	4.7%	5.6%

^1^Transcripts that were grouped into clusters based on shared sequence content.

^2^The percentage of RNA-seq reads that mapped back to the transcriptome assembly.

^3^Non-redundant plant hit descriptions.

^4^Benchmarking Universal Single-Copy Orthologs.

As expected, a significantly longer N50 (1747 vs 433 bases) and higher overall alignment rate (96.1% vs 77.6%) were achieved in the ovule assembly compared with the LCM egg, while the combined LCM egg plus ovule assembly had a slightly lower N50 than ovule alone. By subjecting our transcriptome assemblies to BUSCO analysis^31^, the ovule transcriptome was found to be near-complete, the LCM egg transcriptome highly fragmented and the combined LCM egg plus ovule transcriptome slightly less complete than ovule alone. A BLASTN (e-value < 1e^−10^) search against the NCBI nt database found the greatest number of unique plant hit descriptions (36,861) in the LCM egg plus ovule assembly with slightly fewer (33,826) in the ovule assembly. The fewest unique plant hit descriptions (23,049) were found in the LCM egg assembly. These observations indicate that most LCM egg transcripts are represented in the ovule transcriptome, and that the LCM egg plus ovule assembly not only properly captured the diversity from both individual assemblies but also captured transcripts that failed to be assembled and annotated solely by LCM egg or ovule data alone. For example, *CcASGR-BBML* reads were detected in the apomictic ovule read data but no contig was present in the ovule assembly, whereas a *CcASGR-BBML* contig (*TRINITY_DN28916_c0_g1_i1*) was identified in the LCM egg plus ovule assembly.

The number of contigs in the LCM egg assembly and in the LCM egg plus ovule assembly with detectable egg cell expression (hit count > 0 across all LCM egg libraries) was examined (Table 3). The LCM egg plus ovule assembly showed an additional 14,831 contigs with LCM egg expression (a 9.8% increase) and a 26.8% increase in the number of annotations identified. These results support the combination of reads generated from the LCM egg and ovule to improve both assembly and annotation of egg cell sequences. The LCM egg plus ovule assembly was used for the downstream analyses.

**Table 3:** Assembly and annotation statistics for de novo egg assembly and egg cell-expressing transcripts in LCM egg plus ovule combined assembly.

	LCM egg assembly	LCM egg plus ovule assembly
Number of Trinity contigs expressed in LCM egg reads	151,167	165,998
With annotation by BLASTN against NCBI nt database	47.87%	55.26%

### Cell-type specific expression patterns

The top 50 most abundantly expressed LCM egg transcripts accounted for 12.7% of total LCM egg reads and included constitutively expressed transcripts from mitochondrion, chloroplast, and ribosomal protein genes (Supplementary Table S2). Among the 165,998 transcripts expressed in the LCM egg, 91,730 (55.26%) had hits in the NCBI nt database (e-value < 1e^−10^), and 71,631 of those (78.1%) to a close relative, *Setaria italica* (foxtail millet), consistent with the known evolutionary relationship between these species^32–34^.

**Figure 2:**
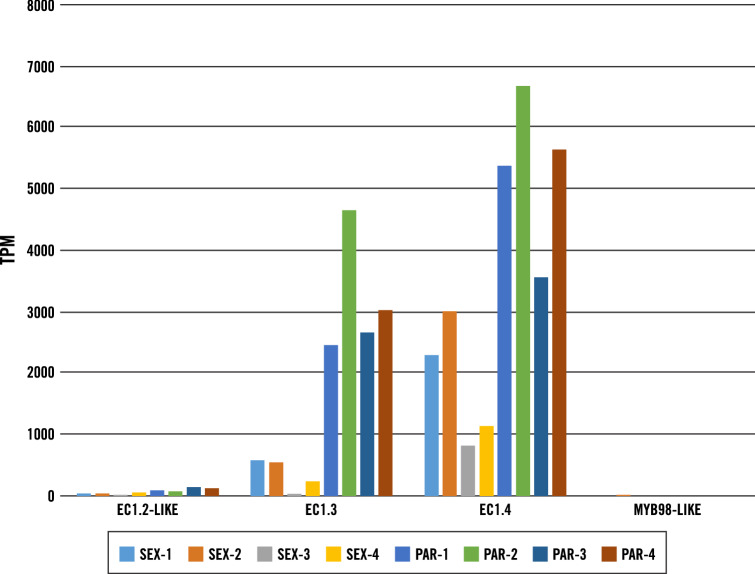
Cell type-specific gene expression plot. This plot is based on TPM of sexual (SEX) and parthenogenetic (PAR) LCM egg read counts.

**Figure 3:**
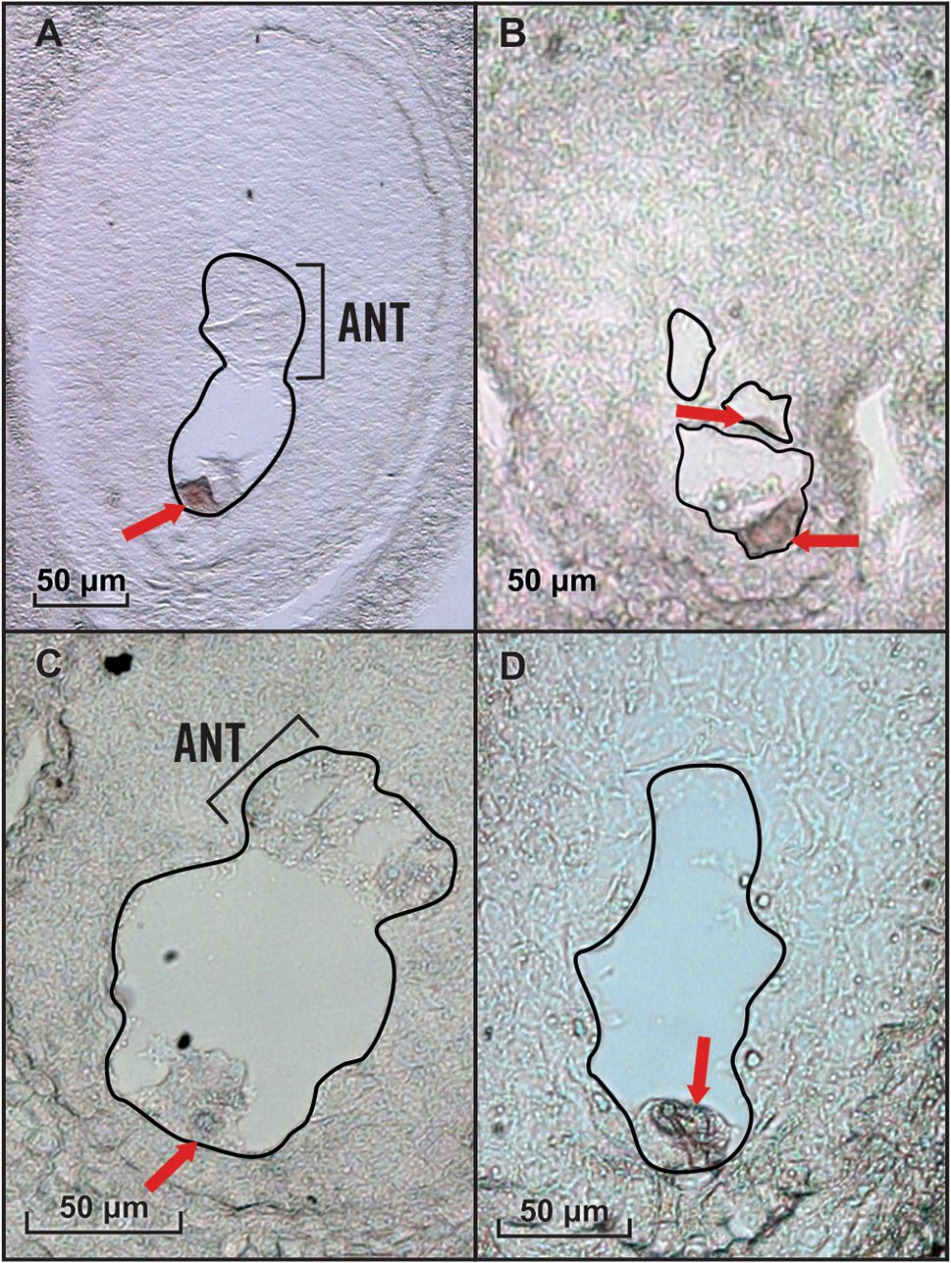
RNA in situ hybridization of sexual (B-2S) and parthenogenetic (B-12–9) ovary sections at day of anthesis. (**A**) A sexual ovary section displaying egg-cell specific signal with an *EC1.3* probe. (**B**) A parthenogenetic ovary section with multiple aposporous embryo sacs, displaying two egg-cell signals with an *EC1.3* probe. (**C**) A sexual embryo sac displaying observed background/sense signal. (**D**) A parthenogenetic ovary section with a single aposporous embryo sac displaying egg-cell *CcASGR-BBML* signal. Black outlines define both sexual and aposporous embryo sacs. Sexual embryo sacs are denoted with brackets defining the antipodals (ANT) which do not develop in aposporous embryo sacs. The red arrow denotes the egg cell.

To further check the fidelity of our transcriptome data, the expression of egg cell-specific gene *EC1* (*egg cell 1*)^35^, synergid predominant gene *MYB98*^36^ and a previously experimentally verified parthenogenesis gene *ASGR-BBM-Like*^12^ was examined. Three potential *EC1* orthologs (*EC1.2-like*, *EC1.3* and *EC1.4*) were expressed in all parthenogenetic and sexual LCM egg libraries (Fig. 2). RNA in situ hybridization experiments using a ssRNA probe designed from the *CcEC1.3* transcript (*TRINITY_DN34886_c2_g1_i5*) further confirmed its egg cell expression specificity in both sexual and apomictic ovary sections (Fig. 3A,B). *MYB98,* identified as one of the top five synergid-enriched transcripts in rice [Log_2_ (synergid/egg cell) = 7.23^37^], was detected at extremely low levels in the LCM egg reads, showing that egg cells likely were preferentially captured during LCM or that synergids in the egg apparatus from apomicts differed in their gene expression pattern from those in sexuals (Fig. 2). *CcASGR-BBM-like* expression was detected in all parthenogenetic LCM egg libraries, and was completely absent from the sexual LCM egg libraries, indicating correctly captured eggs with or without parthenogenesis expression. RNA in situ hybridization experiments with an *ASGR-BBML* ssRNA probe also confirmed expression specifically in the parthenogenetic egg cell (Fig. 3C,D). The expression patterns of *EC1, MYB98-like* and *CcASGR-BBM-like* genes in our data, based on TPM and RNA in situs, correlate with previously published expression patterns^12,37^ and support the highly enriched egg-cell specificity of our LCM egg reads.

### Differentially expressed embryonic transcription factors reveal distinctions between parthenogenetic and sexual eggs

**Figure 4:**
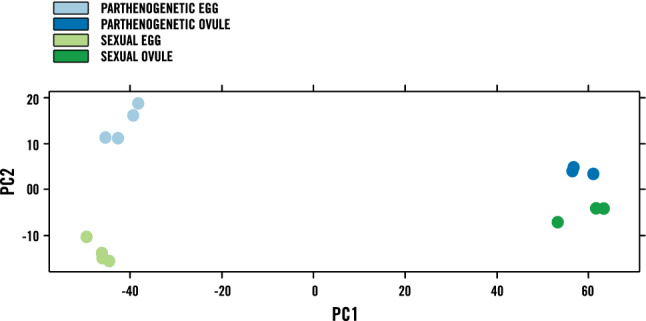
PCA plot of expression profiles across all sequenced samples. The plot is based on the log-transformed CPM (count per million) of read counts (PC1 = 0.4137, PC2 = 0.1032).

**Figure 5:**
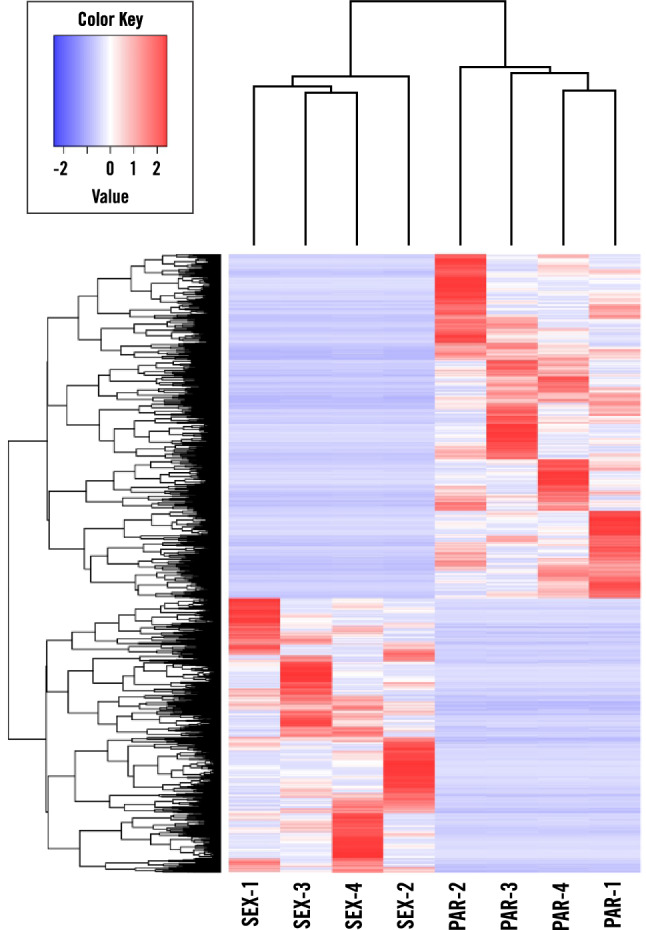
Heatmap of TPM value of DE transcripts. This plot represents every DE transcript (SEX vs PAR) with rows representing each transcript and relative expression (low in blue and high in red).

**Figure 6:**
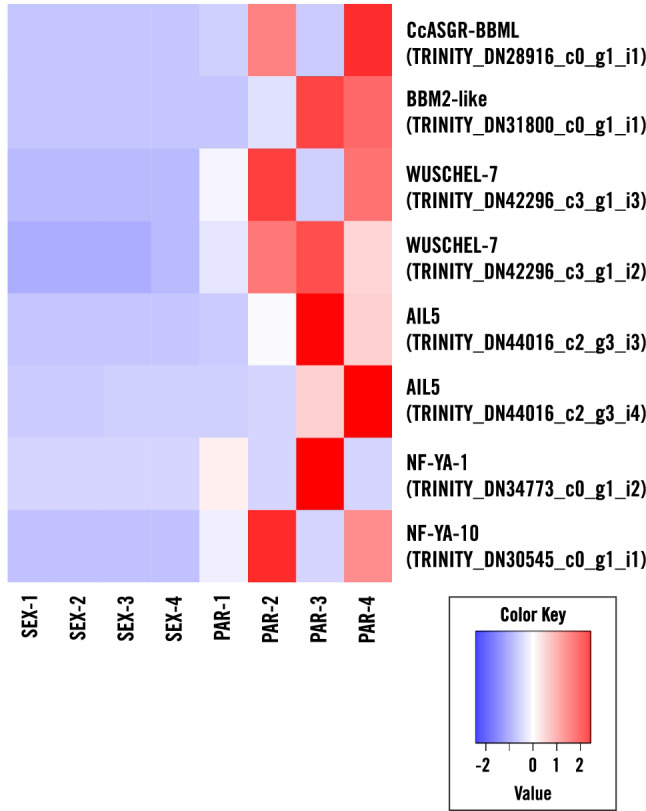
Heatmap of specific transcription factor TPM values. This heatmap represents embryogenic associated transcription factor transcripts with rows representing each transcript (labeled by the gene name and Trinity ID) and relative expression (low in blue and high in red).

The expression profiles across all samples were clustered through principle component analyses with excellent correlation among biological replicates clustering tightly according to genotype and tissue type (Fig. 4). Pairwise comparisons of parthenogenetic LCM egg reads to sexual LCM egg reads were performed to identify differentially expressed genes (DEGs) as those with log_2_FC > 2, false discovery rate (FDR) < 0.05 using DESeq2^38^. Of the 4,625 differentially expressed Trinity transcripts (Supplementary Table S3), 2,571 were up-regulated in the parthenogenetic egg, illustrated in a heat map that includes all the differentially expressed transcripts in parthenogenetic eggs relative to sexual eggs (Fig. 5). Several embryogenesis-related transcription factors were significantly expressed in the parthenogenetic eggs compared to sexual eggs (Fig. 6). Parthenogenetic up-regulated DEGs were further subjected to gene ontology enrichment analyses which identified 175 over-represented GO terms (Supplementary Table S4) including terms such as embryo development (GO: 0,009,790), reproductive process (GO: 0,022,414), transcription factor activity and transcription factor binding (GO: 0,000,989). In a similar enrichment of GO-terms for up-regulated genes in a comparison of parthenogenetic eggs from *Boechera* and sexual eggs from *Arabidopsis*^39^, the only category in common with our results was for regulation of transcription, DNA-templated (GO:0,006,355). This difference could be attributed to developmental differences between apomictic eudicot *Boechera* and the apomictic monocot *Cenchrus* where parthenogenesis in *Boechera* is repressed in the absence of central cell fertilization^40^.

**Figure 7:**
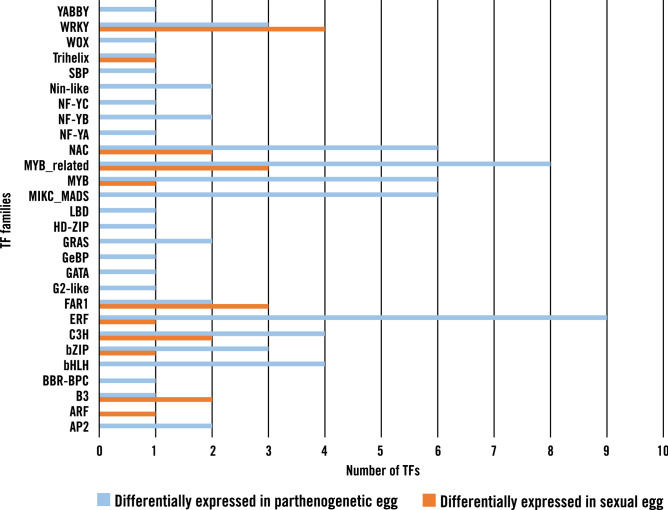
De novo expressed transcription factor families between sexual and parthenogenetic egg.

To assess major regulatory distinctions between parthenogenetic and sexual egg transcripts, LCM egg plus ovule transcripts were subjected to a BLASTX (e-value < 1e-10) search against the plantTFDB (Plant Transcription Factor Database^41^). The search identified 72 potential transcription factors spanning 27 families de novo expressed in parthenogenetic eggs and 21 potential transcription factors spanning 11 families de novo expressed in sexual eggs (Fig. 7; Supplementary Table S5). The AP2 and WOX TF families were among 16 TF families that were exclusively de novo expressed in parthenogenetic eggs. These gene families have been shown to play major roles in embryogenesis and embryo development in monocots and eudicots^42,43^. Using microarrays, a fertilization-induced WOX gene was identified in rice zygotes 2-3 h post-pollination^44^. From RNA-seq data, a comparison of transcription factor families up-regulated in maize zygotes 12 h after pollination compared with maize eggs showed considerable overlap with those up-regulated in *C. ciliaris* parthenogenetic versus sexual eggs, namely AP2, bHLH, bZIP, C3H, GRAS, Homeobox (WOX), MADS, MYB, NAC, Trihelix, WRKY, YABBY, and ZF-HD^25^. At least for transcriptional regulation, this supports our hypothesis that parthenogenetic eggs resemble early stage zygotes that have initiated the maternal to zygotic transition^25,45^.

As expected, an *ASGR-BABY BOOM-like* (*ASGR-BBML*) gene was de novo expressed in parthenogenetic eggs. Expression of this apomixis-locus derived gene induces parthenogenesis in unfertilized eggs of sexual pearl millet, rice and maize in the absence of pollination^12,13^. Ectopic overexpression of *Brassica BBM* induces somatic embryogenesis in *Arabidopsis*^46^ and the rice *BBM1* (LOC_Os11g19060.1) transgene, expressed from an egg-cell-specific promoter, induces embryogenesis in rice eggs without fertilization^47^. Native *OsBBM1* expression in rice was detected in zygotes at 2.5 h after pollination (HAP) (corresponding to karyogamy) in a male-origin-specific manner, and ectopic expression in egg cells under an egg-cell-specific promoter induced parthenogenesis. Differential gene expression analysis also provided evidence for de novo expression of a *C. ciliaris BBM2-like* gene (*TRINITY_DN31800_c0_g1_i1*) in unfertilized parthenogenetic LCM eggs. The parthenogenesis gene *CcASGR-BBML* was de novo expressed at very low levels (average TPM = 3.7) in parthenogenetic eggs, while the *CcBBM2-like* gene was also de novo expressed in parthenogenetic eggs with relatively abundant expression (average TPM = 44.6). No read counts were identified for the *CcBBM2-like* transcript in sexual egg sequences. Based on the observation that *BBM* has a transcriptional auto-activation nature^48^, we speculate that *CcBBM2* expression may be due to *CcASGR-BBML* activation and *CcBBM2* auto-activation. The parthenogenesis pathway may be initiated by *CcASGR-BBML* and proceed through its activation of *CcBBM2* together with *CcBBM2* auto-activation to further promote cell proliferation and embryogenesis.

*C. ciliaris WUSCHEL-related homeobox 7* (*WOX7*) genes (*TRINITY_DN42296_c3_g1_i3 and TRINITY_DN42296_c3_g1_i2*) showed de novo and up-regulated expression in parthenogenetic eggs, respectively. Over-expression of maize *BBM* (orthologous to the *C. ciliaris BBM2-like* gene) and *WUSCHEL2* in somatic tissues of maize, sorghum, rice and sugarcane greatly increases embryogenic potential thereby enhancing transformation frequency^49^. It is possible that the de novo-expressed and up regulated *WUSCHEL* from parthenogenetic eggs works in concert with *BBM2* to promote parthenogenesis. It is noteworthy that *BBM* and *WUSCHEL* were de novo expressed in rice zygotes, 2.5 h after pollination at karyogamy^24^ suggesting a role in promoting zygotic embryogenesis. *WUSCHEL* is thought to be required for maintaining meristem identity^50^ and its overexpression can induce organogenesis and somatic embryogenesis in shoot and root tissues^42,51^. Several genes overlapped between our DEGs and *WUSCHEL*-target genes^52^ (Supplementary Table S6) but none of them plays a central role in embryogenesis suggesting that some of the *WUSCHEL*-mediated transcriptional change may be ancillary to regulating parthenogenesis or that another set of embryogenesis-related targets is specific to *CcWOX7*.

Besides *CcBBM2* and *CcWOX7*, embryonic factor *CcAINTEGUMENTA-LIKE 5* (*AIL5*) gene (*TRINITY_DN44016_c2_g3_i3* and *TRINITY_DN44016_c2_g3_i4)* showed de novo and up-regulated expression in parthenogenetic LCM egg. Ectopic expression of *Arabidopsis AIL5* can induce somatic embryo formation in *Arabidopsis*^53^. The identification in parthenogenetic eggs of up-regulated and de novo expressed embryonic transcription factors that previously have been shown to function in parthenogenesis, somatic embryogenesis, or zygotic embryogenesis, demonstrated the utility of our data and confirmed that embryogenic potential is one of the major functional distinctions between parthenogenetic and sexual eggs.

### Transcriptional change in parthenogenetic eggs may confer the core parthenogenesis pathway

Based on prior research that the transcription factor *ASGR-BBML* is the only experimentally verified apomict-derived parthenogenesis gene^12,13^, and that *BBM* transcription factors are known to function with other proteins to control cell proliferation and somatic embryogenesis^48,54,55^, we hypothesize that the core natural parthenogenesis pathway initiated by *ASGR-BBML* may advance through interactions with other TFs followed by up- or down-regulation of their target genes.

Candidate BBM-target genes that were directly activated by BBM expression in *Arabidopsis* seedlings and those with DNA sites bound by BBM in *Arabidopsis* somatic embryos through chromatin immunoprecipitation sequencing (ChIP-seq)^48,54,55^ were examined. Overlap between these previously reported genes and our differentially expressed genes was found. Putative orthologs were identified through BLASTN description annotation (NCBI nt database) similarities and/or sequence similarities through BLASTX search using full length contigs (their corresponding best hit in NCBI nt database) against Araport11_genes.201606.pep (1e^−5^) (Supplementary Table S7). These included *BBM* and *AIL5* described above. In addition, potential orthologs (*TRINITY_DN34773_c0_g1_i2* nuclear transcription factor Y subunit A-1 and *TRINITY_DN30545_c0_g1_i1* nuclear transcription factor Y subunit A-10) of CcNUCLEAR TRANSCRIPTION FACTOR Y SUBUNIT A-9, similar to another *BBM* target gene thought to play a role in inducing embryogenesis when overexpressed in *Arabidopsis*^56^, were upregulated in parthenogenetic eggs suggesting that *BBM* may also interact with *NF-YA* to control embryogenesis.

Apart from the parthenogenetic egg up-regulated transcripts with similarity to *BBM*-target genes that correspond to genes with known function to induce embryogenesis, those that may play roles in transcription, signaling, protein–protein interaction and cytoskeleton organization that function in embryogenesis also were identified. For example, two members of a well-studied gene family *ACTIN DEPOLYMERIZING FACTOR* (*ADF*), *ADF2* (*TRINITY_DN36541_c0_g1_i1*, Log_2_FC = 6.34, FDR = 0.03, average TPM in parthenogenetic egg = 40.03) and *ADF5* (*TRINITY_DN37217_c0_g3_i4*, Log_2_FC = 8.75, FDR = 0.001, average TPM = 12.35), thought to control actin dynamics^57^ and reorganize the actin cytoskeleton under *BBM* activation^48^, were up-regulated in parthenogenetic eggs.

### Histone modification and chromatin remodeling factors are differentially expressed in parthenogenetic vs sexual eggs

The chromosomal regions associated with apomixis in *C. ciliaris* are largely heterochromatic^8^, suggesting potential for epigenetic regulation. Comparative small RNA-seq between sexual and apomictic spikelets of *Paspalum notatum* provided evidence that RNA-dependent regulation of auxin pathways may be important for the expression of apomixis^20^. A comparative transcriptomics study between sexual and apomictic ovule nucellus at a pre-meiotic developmental stage in *Hypericum perforatum* L. showed that RNA-mediated DNA methylation, histone and chromatin modifications were associated with aposporous apomixis expression^19^. Moreover, a methylation status analysis of the apomixis-specific region in *Paspalum* spp. suggests a possible epigenetic control of parthenogenesis, where the factors controlling repression of parthenogenesis might be inactivated in apomictic *Paspalum* by DNA methylation^58^.

Chromatin states are mainly determined via two mechanisms: covalent modifications of histones and DNA methylation^59^. Major histone modification events can be categorized into acetylation, methylation, phosphorylation and ubiquitination^60^, which affect chromatin states resulting in activation or repression of gene expression^61–63^. In plants, studies have shown that histone acetylation/deacetylation and histone methylation play a fundamental role in regulating plant development.

Among DEGs between parthenogenetic and sexual eggs, multiple histone deacetylase genes (*TRINITY_DN41443_c1_g1_i1, TRINITY_DN39020_c3_g1_i2, TRINITY_DN37516_c2_g2_i6, and TRINITY_DN42464_c1_g1_i7*) were exclusively up-regulated in parthenogenetic eggs while a single putative MYST histone acetyltransferase gene (*TRINITY_DN38730_c1_g1_i7)* was up-regulated in sexual eggs. Hypoacetylation is known to be associated with chromatin condensation and transcriptional suppression^63^, which suggests that there are some genomic regions in parthenogenetic eggs that are hypoacetylated and genes within those regions may be transcriptionally silent. Alternatively, evidence is emerging in plants for a balance of acetylation/deacetylation for gene activation^64^. The short fruit phenotype in cucumber results from a mutation in *Histone deacetylase complex1* (*HDC1*; *SF2* in cucumber), a gene whose product interacts with multiple histone deacetylases to enhance expression of cell cycle, DNA replication, and chromatin assembly genes and promote cell division, but also to repress expression of suppressors of auxin, gibberellin, and cytokinin biosynthesis and responses. Limited histone 3 acetylation in gene bodies and properly acetylated promoters and enhancers were shown to promote transcriptional elongation, at least in mammalian cells^65^.

Another potential role for histone deacetylases is to allow methylation of the H3K9 residue^66,67^ and interaction with DNA methyltransferase to repress gene expression^68^. Multiple histone methylation factors were differentially expressed, most being up-regulated in parthenogenetic eggs. Among these were *Cchistone-lysine N-methyltransferase ATXR4* (*TRINITY_DN34478_c0_g1_i3*), *Cchistone-lysine N-methyltransferase TRX1* (*TRINITY_DN46041_c2_g1_i2*), *CcH3 lysine-9 specific SUVH1* (*TRINITY_DN42267_c0_g1_i1*), and *CcH3 lysine-9 specific SUVH4 (KRYPTONITE)* (*TRINITY_DN46013_c1_g1_i6*). *SUVH4* encodes a protein that methylates H3K9, is required for the maintenance of cytosine methylation in the CpNpG context, and represses retrotransposon activity in *Arabidopsis*^69^. Another parthenogenetic up-regulated histone-lysine N-methyltransferase gene, *CcH3 lysine-9 specific SUVH1* shows similarity with a gene in *Arabidopsis* that is associated with heterochromatic H3K9 dimethylation, and may play a role in heterochromatin gene silencing^70^. Apart from histone modification, DNA methylation is also involved in determining chromatin states. One of the best characterized classes of DNA methylation genes in *Arabidopsis*, *DOMAINS REARRANGED METHYL TRANSFERASE2* (*DRM2*), responsible for de novo DNA methylation in all sequence contexts and mediation of transgene silencing^71^, was represented by one *CcDRM2* gene (*TRINITY_DN36171_c0_g1_i3*), de novo expressed in parthenogenetic eggs. Another *CcDRM2* gene (*TRINITY_DN46376_c0_g4_i1*) was up-regulated in sexual eggs. The de novo expression of one *CcDRM2* gene in parthenogenetic eggs and the up-regulation of another in sexual eggs implies that increased de novo DNA methylation may be common to both parthenogenetic eggs with parthenogenetic fate and sexual eggs with embryogenesis fate upon fertilization. This is consistent with the observation that embryogenesis is characterized by increased de novo DNA methylation^72^. However, it is not known if these two *CcDRM2* genes function somehow to differentiate sexual and parthenogenetic egg cell fate through de novo DNA methylation.

Hypoacetylation and H3K9 methylation activities suggest enhancement of repressive chromatin states in parthenogenetic eggs even though they are transitioning to an active stage of cell division. Specific chromatin regions may be repressed in parthenogenetic eggs while others are derepressed in order to release totipotency and achieve competency to initiate parthenogenesis. Day0 unpollinated parthenogenetic eggs are biologically active in terms of having the capacity to form embryos two days after anthesis and are transcriptionally active as indicated by GO enrichment analyses reflecting the active side of chromatin states. Several Jumonji-like proteins were up-regulated in parthenogenetic eggs including JMJ25-like (*TRINITY_DN44176_c1_g1_i3*; *TRINITY_DN46670_c1_g4_i1*) which demethylates H3K9, regulating gene expression through epigenetic modifications^73,74^. Histone 3 lysine 9 methylation homeostasis may be regulated by the interplay of Jumonji and SUVH proteins. Histone 3 lysine 4 methylation, positively associated with actively transcribed genes^75^, was indicated by a COMPASS-like H3K4 histone methylase component *WDR5B* gene and a H3K4 histone-lysine N-methyltransferase *CcTRX1* gene, both up-regulated in parthenogenetic eggs. Similar genes were shown to activate flowering under long day conditions^76,77^. A rice ortholog was shown to promote flowering by interacting with a DNA-binding C2H2 zinc finger protein SIP1 to activate *EARLY HEADING DATE 1*, a B-type response regulator^78^. Although the histone-lysine N-methyltransferase *ATXR4* was up-regulated in parthenogenetic eggs, no direct evidence is available to infer its function. In *Arabidopsis ATX1*, *ATX2*, *ATXR3*, and *ATXR7* positively regulate *FLC* via H3K4 methylation^79–82^ while *ATXR5* and *ATXR6* control the heterochromatin condensation and heterochromatic elements silencing via methylation of H3K27^83^. Taken together, the differential expression of histone deacetylases and histone methylases as well as a de novo expressed DNA methyltransferase is likely to both repress and activate a spectrum of genes that characterize parthenogenetic eggs assuming a parthenogenesis fate.

### Cell cycle

The idealized cell cycle is comprised of four successive phases: G1 (Gap 1), S (Synthesis), G2 (Gap 2) and M (Mitosis), through which parthenogenetic egg cells must traverse as they become competent for cell division. The expression profiles of some key cell-cycle regulators were examined in an attempt to identify the cell cycle stages for Day 0 parthenogenetic and sexual egg cells.

A *CcBREAST CANCER SUSCEPTIBILITY 1* (*BRCA1)* transcript (*TRINITY_DN29461_c0_g1_i1*) was up-regulated in parthenogenetic eggs. This gene plays a role in the G2/M checkpoint, particularly upon detection of DNA damage, limiting the proliferation of cells containing replication defects^84,85^. Similarly, another gene, *CcSUPPRESSOR OF GAMMA RESPONSE 1* (*SOG1*) (*TRINITY_DN39490_c0_g1_i2*), that responds to DNA damage signals, and whose phosphorylated form controls several cell cycle and DNA damage repair genes^86^, was up-regulated in parthenogenetic eggs. BRCA1 has been identified as a target of SOG1, but was uniquely up-regulated in parthenogenetic eggs compared with other common targets. Since the expression of *BRCA1* was barely detectable in the sexual eggs, the parthenogenetic egg might have reached the G2/M checkpoint while the sexual egg remained at a stage prior to G2. Aside from a potential cell cycle marker, it is tempting to speculate that an apomict undergoing many cycles of clonal reproduction through seeds may have adapted gene expression to ensure DNA repair in somatic cell-derived eggs destined to undergo parthenogenesis.

### Auxin and calcium

Auxin plays a role in virtually every aspect of plant growth and development including early embryogenesis^87^ and gamete specification in the female gametophyte^88^. In relation to apomixis, auxin treatment of the inflorescence of apomictic *Poa pratensis* was used to rapidly and reliably test whether the egg cell was competent for parthenogenesis, showing that exogenously supplied auxin promoted the expression of apomictic parthenogenesis^89^.

Among DEGs, an auxin receptor *CcTRANSPORT INHIBITOR RESPONSE 1* (*TIR1*) gene (*TRINITY_DN40331_c0_g1_i1*)^90–92^ was up-regulated in parthenogenetic eggs indicating that the parthenogenetic egg may differ from the sexual egg in auxin perception. Interestingly, auxin negative regulators *CcAUX/IAA* genes (*IAA17*, *TRINITY_DN37013_c0_g1_i2; IAA2, TRINITY_DN44579_c0_g3_i1; IAA31, TRINITY_DN39140_c0_g1_i3; IAA29, TRINITY_DN32631_c0_g1_i2*) were exclusively up-regulated in parthenogenetic eggs. *AUX/IAAs* are thought to interact with SCF^TIR1^ and lead to ubiquitination and degradation through the 26S proteasome in the presence of high auxin concentrations, derepressing auxin response factors (ARFs) to allow ARFs to transcriptionally activate or repress downstream auxin responsive genes^93,94^. Interestingly, 10 *BTB/POZ*-domain transcripts were up-regulated in parthenogenetic eggs versus only one in sexual eggs, suggesting an active proteasomal ubiquitin-mediated degradation pathway in parthenogenetic eggs. At least one of these *BTB/POZ*-domain transcripts contains an ankyrin repeat domain similar to BLADE ON PETIOLE (BOP) lateral organ boundary genes^95^. The ankyrin repeat interacts with basic Leu repeat transcription factors^96^ for which six transcripts were up-regulated in parthenogenetic eggs. These up-regulated genes suggest a regulatory network in parthenogenetic eggs involving a dynamic transcriptional and protein turnover response to hormones shaping growth and development. Induction of auxin pathway genes in maize zygotes 12 h after pollination was also observed^25^.

Crosstalk between auxin and ethylene signaling pathways leading to cooperative regulation of developmental processes has been documented^97^. *ETHYLENE INSENSITIVE 2* (*EIN2*; *TRINITY_DN47673_c2_g1_i1*) along with 14 ethylene responsive transcription factors were up-regulated in parthenogenetic eggs versus only one in sexual eggs. Among these transcription factors were the embryogenesis related genes already discussed, *BBM2* and *AIL5*, but also other *AP2*-domain containing, ethylene responsive genes *CYTOKININ RESPONSE FACTOR* (*CRF1*; *TRINITY_DN40560_c0_g1_i1*), *ABA INSENSITIVE* (*ABI4*; *TRINITY_DN49291_c1_g1_i1*), *RELATED TO APETALA2* (*RAP2*; *TRINITY_DN39096_c2_g1_i2*; *TRINITY_DN46047_c0_g3_i2*), *ERF61* (*TRINITY_DN42510_c2_g1_i1*), *ERF73* (*TRINITY_DN40591_c1_g2_i1*), and *WAX INDUCER 1* (*WIN1*; *TRINITY_DN42753_c2_g1_i3*). These transcription factors may integrate multiple hormone responses.

Calcium ionophore is reported to stimulate parthenogenesis in mouse oocytes^98^ but calcium alone is not sufficient to induce parthenogenesis in plants. As for plants, Ca^2+^ changes in egg cells are widely thought to be associated with successful fertilization and egg activation^99–101^. In our data, intracellular calcium receptor protein genes, calmodulin (*TRINITY_DN44663_c0_g1_i2*; *TRINITY DN33744_c3_g1_i1; TRINITY_DN34327_c1_g1_i3*)^102^ and its effector calcium/calmodulin-dependent protein kinases (*TRINITY_DN44558_c1_g2_i1*; *TRINITY_DN40595_c1_g3_i1*)^103^, were exclusively up-regulated in parthenogenetic eggs compared to the sexual eggs, suggesting the presence of an internal Ca^2+^ increase and calcium-triggered signaling pathway in parthenogenetic eggs. This may reflect some aspects of parthenogenetic egg activation but its correlation to parthenogenesis is still unknown.

With the differentially expressed genes and their annotations generated in this study, the potential core pathways diagnostic of natural parthenogenesis in an apomictic grass species and pathways that may play an ancillary role in promoting parthenogenesis based on sequence similarities to previously identified genes and pathways were discussed. However, to experimentally test our hypotheses, chromatin immunoprecipitation-seq (ChiP-seq) on transcription factor-bound DNA would be essential to study target genes or protein-DNA interactions. Yeast two hybrid experiments may be needed to experimentally investigate physical interactions between proteins encoded by differentially expressed genes or other embryogenic factors identified in this study. Bisulfite- or ATAC(Assay for Transposase Accessible Chromatin)-sequencing could potentially be used to ultimately check the chromatin state differences between sexual and parthenogenetic egg cells, but both would be challenging at a single cell level in plants. Progress recently has been made to apply single nucleus Assay for Transposase Accessible Chromatin sequencing (sNucATAC-seq) technologies to decipher cell-type-specific pattern of chromatin accessibility in Arabidopsis roots^104^. As an initial step, this study has provided numerous sequence and computational analyses for sexual and parthenogenetic eggs, which are valuable for developing testable hypotheses to further explore natural parthenogenesis in grasses.

## Materials and methods

### Plant material and floret collection

The *C. ciliaris* plants used as source materials were vegetatively propagated tillers of the sexual genotype B-2s and natural apomictic genotype B-12–9^105^. Plants were grown in the greenhouse (24–30 °C) for head collection from July to September in 2016. Heads were bagged prior to stigma exsertion and stigmas were manually removed with tweezers upon appearance. The heads remained bagged until the day of anther exsertion (anthesis) at which time florets were collected from 8:00–11:00 am with fresh anthers half or fully exserted and immediately fixed in Farmer’s fixative (ethanol:glacial acetic acid, 3:1)^106^ and stored at 4 °C for overnight. Fixed florets were then transferred and stored in 70% ethanol (DEPC-treated water) at 4 °C.

### Ovary clearing and microscopic observation

For ovary clearing, stigma-free heads were collected on the day of (Day0) and two days after (Day2) anthesis and immediately fixed in Farmer’s fixative. Ovaries were dissected from florets, and dehydrated in a graded ethanol series for two hours in each step as follows: 70% ethanol, 85% ethanol, and 100% ethanol, followed by another 100% ethanol incubation overnight. Ovaries were transferred to a methyl salicylate series for 2 h in each step as follows: ethanol: methyl salicylate (3:1), ethanol: methyl salicylate (1:1), ethanol: methyl salicylate (1:3), and followed by a 100% methyl salicylate incubation overnight^107^. Cleared ovaries were observed under DIC (differential interference contrast) microscope (Supplementary Fig. S1).

### Ovule collection, RNA extraction, cDNA Synthesis, amplification, library construction and sequencing

Intact ovaries were dissected from fixed florets on ice using fine tweezers to avoid physical damage. Ovules were subsequently isolated by pulling apart the bifurcated stigma to separate the ovary wall from the ovaries. Fifty ovules per sample were collected. Ovule RNA was extracted using RNeasy Plant Mini Kit (QIAGEN) following the manufacturer’s protocol. The quantity and quality of RNA were checked using the Qubit 2.0 Fluorometer RNA assay (Invitrogen) and an Agilent 2100 Bioanalyzer using a RNA 6000 nano kit (Agilent Technologies), respectively. For each sample, 500 ng of total RNA was used for poly-A capture, cDNA synthesis and amplification using KAPA stranded RNA-seq Kit. Libraries were quality checked with the Qubit 2.0 Fluorometer dsDNA high sensitivity assay (Invitrogen) and Fragment Analyzer Automated CE System (Agilent Technologies), and were sequenced on an Illumina NextSeq (300 Cycles) PE150 Mid Output flow cell on which three biological replicates of two genotypes were pooled.

### Tissue preparation for laser capture microdissection

Intact ovaries were dissected on ice from fixed florets using fine tweezers to avoid physical damage and were dehydrated in a graded ethanol series on ice with gentle shaking for 20 min in each step as follows: 80% ethanol, 90% ethanol, and three changes of 100% ethanol. Ovaries were stored overnight in 100% ethanol at 4 °C, and transferred the next day to a xylene series with gentle shaking for 20 min in each step as follows: ethanol: xylene (3:1), ethanol: xylene (1:1), ethanol: xylene (1:3), and three changes of 100% xylene. Xylene-cleared ovaries were transferred to a Paraplast series in an incubator (54 °C) with shaking for 9 h in each step as follows: xylene and Paraplast (1:1) mixture and seven changes of 100% Paraplast. Paraplast-infiltrated ovaries were embedded in Paraplast blocks using a tissue embedding center, and 8-µm sections were cut on a rotary microtome (Leica RM2145, Germany). Sections were floated on methanol and mounted on metal frame PET foil slides (Leica) that had been UV-irradiated (DNA transfer lamp, Fotodyne) for 30 min. Slides were heated on a slide warmer at 42 °C overnight to air-dry and stretch the Paraplast ribbons. Prior to egg identification and laser capture microdissection, PET foil slides with ovary sections were de-paraffinized in xylene for two changes of 5 min each with gentle shaking and air-dried for 1 h in a fume hood. A few ovary sections were stained with Safranin and FastGreen to check tissue quality, and the egg cell was identified under a microscope (Fig. 1). Unstained sections were used for LCM.

### Laser capture microdissection and egg cell collection

A Leica LMD6000 laser microdissection system was used to capture the egg cell from tissue sections prepared as above. Before loading PCR tubes with cap into the collection device, 8 μl of RNAlater was pipetted into the lid of the cap. Microdissections were then performed by drawing a line or circle around the egg along which the laser beam cut (Supplementary Fig. S2), with the laser setting as follows: Power 45, Aperture 4, Speed 9 and Specimen balance 15. After the LCM, the egg sections fell into the lid by gravity, were preserved by the RNAlater, and stored at −80 °C until use. Around 1000 egg sections (from 500 ovules) per biological replicate (4 B-2s and 4 B-12–9) were collected.

### Egg RNA extraction, cDNA Synthesis, amplification, library construction and sequencing

Egg RNA was extracted using RNeasy Plus Micro Kit (QIAGEN) following the manufacturer’s protocol with minor modifications. The quantity and quality of RNA were checked using the Qubit 2.0 Fluorometer RNA assay (Invitrogen) and an Agilent 2100 Bioanalyzer using an RNA 6000 Pico kit (Agilent Technologies), respectively. For each of the samples, 2 ng of total RNA was used for cDNA synthesis and amplification using SMART-Seq v4 Ultra Low Input RNA Kit (Clontech) with modifications. Modifications included increasing the elution buffer volume, resuspension time, constantly mixing the cDNA-bound beads as well as increasing cDNA amplification cycles to improve the cDNA recovery and yield. The concentration and profile of the amplified cDNA were checked using the Qubit 2.0 Fluorometer dsDNA high sensitivity assay (Invitrogen) and the Fragment Analyzer Automated CE System (Agilent Technologies), respectively. For each sample, 100 ng of cDNA was sheared using a Covaris E220 Evolution with 350 bp insert setting and was used for library construction with the TruSeq DNA Nano LT Kit (Illumina) following the manufacturer’s suggested protocol with modifications mainly by doing twice the Enrich DNA Fragments step. Libraries were quality checked with the Qubit 2.0 Fluorometer dsDNA high sensitivity assay (Invitrogen) and Fragment Analyzer Automated CE System (Agilent Technologies), and were sequenced in two separate runs of NextSeq (300 Cycles) PE150 Mid Output flow cell on which two biological replicates of each genotype were pooled. Further details of modified protocols can be found in Supplemental Experimental Procedures.

### Data preprocessing

Both ovule and egg raw reads were cleaned by removing adapter sequences, over-represented technical sequences detected by FASTQC^108^, and sequences of poor-quality using Trimmomatic^109^. For LCM egg sequencing data, 5–7 bases from the 5′ and 3′ ends of the trimmed reads were further cut using TrimGalore^110^ to avoid potential sequence bias. Comprehensive rRNA removal was done using SortMeRNA^111^. To further identify and remove potential biological contaminants, a de novo assembly was constructed with cleaned reads from the eight LCM egg libraries using Trinity^112,113^. Trinity contigs were first annotated by a BLASTN^114^ search against the NCBI nt database (e-value cutoff of 1e^−10^). Using these annotations, a custom non-plant contamination database was constructed by extracting the aligned portion of the subject sequences from the contigs that hit (< 1e^−10^) bacterial, fungal and animal sequences stored in the NCBI nt database. The non-plant contamination database was indexed as the reference to clean each library until no significant amount of non-plant hit was seen in the final assembly. All cleaned reads were used for downstream analyses.

### De novo trinity assembly

Trinity-2.8.4.simg was used for de novo transcriptome assembly. For the ovule plus egg transcriptome, cleaned egg and ovule reads (Supplementary Table S1) were combined into a single fastq file, assembled using Trinity (parameters –seqType fq –single –run_as_paired –max_memory 150G –CPU 8 –no_version_check –normalize_reads). Egg, ovule assembly was constructed using solely the cleaned egg, cleaned ovule reads respectively.

### Trinotate annotation and gene ontology enrichment analyses

Trinity transcripts were annotated with Trinotate through a BLASTX^114^ search (e-value = 1e^−5^) against a comprehensive protein database comprised of the Swiss-Prot^115^ and UniRef90 protein databases. Putative coding regions within each Trinity transcript were predicted using TransDecoder (http://transdecoder.github.io), and the predicted coding sequences were further annotated through BLASTP (e-value = 1e^−5^) alignment against the comprehensive protein database mentioned above and for protein domains search using hmmer (http://hmmer.org/) and PFam^116^. SignalP^117^ was used to predict the potential signal peptides in the transcripts. All results were integrated by Trinotate, stored in an SQLite database, and then reported as a tab-delimited excel file (Supplementary Table S8). The functional enrichment analyses were done on significantly differentially expressed transcripts set against the whole expressed transcripts using Trinotate-assigned GO annotations and GOseq^118^, and enriched GO terms of parthenogenetic up-regulated transcripts were reported as a tab-delimited file.

### BUSCO completeness analyses

The completeness of the transcriptome assembly was examined by subjecting the Trinity.fasta file to BUSCO^31^ analyses using the command run_busco -i Trinity.fasta -l liliopsida_odb10 -m tran.

### Transcript abundance estimation and differential expression analysis

Kallisto^119^ software was used to quantitate the expression level for transcripts, and DESeq2^38^ was used to identify significantly differentially expressed transcripts.

### Plant transcription factor identification

The potential plant transcription factors in the trinity transcripts were identified by BLASTX search (e-value < 1e^−10^) against a close relative’s, *Setaria italica*, transcription factor database downloaded from http://planttfdb.cbi.pku.edu.cn/download.php^41^.

### *RNA *in situ

Tissue for RNA in situs were prepared and sectioned according to the LCM protocol. Sections were floated on methanol and mounted on microscope slides (Fisherbrand Probe On Plus). Slides were heated on a slide warmer at 42 °C for overnight. Slides were de-paraffinized in xylene for 10 min twice, followed by a hydration series: 2 times of 100% ethanol, 95% ethanol, 70% ethanol, 50% ethanol, 30% ethanol, and 2 times of DEPC-treated water (2 min for each). Rehydrated slides were incubated in 0.2 M HCl for 10 min, and then washed in DEPC-treated water, 2 times of 2 × SSC (saline sodium citrate), then DEPC-treated water, 5 min each. Slides were then treated with a mixture of 100 mM Tris pH 8.0, 50 mM EDTA pH 8.0 and 1 μg/mL proteinase K at 37 °C for 20 min, followed by a wash in PBS (phosphate buffered saline) for 2 min. Slides were incubated in 2 mg/mL glycine in PBS for 2 min to block proteinase K, and washed in PBS for 30 s twice. Slides were fixed in 4% formaldehyde in PBS for 10 min and washed in PBS for 5 min twice, then dehydrated as follows: 2 times of DEPC-treated water, 30% ethanol, 50% ethanol, 70% ethanol, 95% ethanol and 2 times of 100% ethanol (2 min each). Slides were dried for 15 min in the fume hood.

### Probe synthesis

The primers 5′-GGCGAGATCATCCTGTACCT-3′ and 5′-GTCGCAGTATCCCTTGAGCAT-3′ were designed based on the *EC1.3* (egg cell-specific 1.3) Trinity transcript. The primers 5'-TTTAGCTGCTCTCAAGTACCGG-3' and 5'-CCTGGTAACCCCTCGGTAAATT-3' were designed from the *PsASGR-BBML* cDNA sequence (GenBank: EU559277.2). The amplicons were cloned into pGEM-T Easy Vector Systems (Promega), sequenced through Macrogen, and reverse transcribed into ssRNA probes using the MAXIscript SP6/T7 Kit (Invitrogen).

### Hybridization

Hybridization buffer was made with each of the following components at a final concentration of: 50% HiDi formamide (Applied Biosystems),100 μg/ml Yeast tRNA (Invitrogen), 0.75% blocking reagents (Roche), 2.5% dextran sulfate (Millipore), 2.5 mM EDTA and DEPC-treated water to make up for the total volume. Probe (0.1 μl) was added to 10 μl of hybridization buffer and heated at 80 °C for 3 min. 190 μl hybridization buffer was added to 10 μl pre-denatured probe-hybridization buffer mixture and kept at 55 °C until use. For each slide with pretreated ovary sections, 200 μl pre-denatured probe-hybridization mixture were added, and sealed with another microscope slide on top. The slide pairs were incubated in a humidified environment at 50 °C for overnight.

### Antibody reaction and detection

After incubation for hybridization, slides were first washed in 2 × SSC (Sigma-Aldrich) and 50% formamide (Sigma-Aldrich ACS reagent) for 1 h at 50 °C each, followed by another wash in TBS buffer (100 mM Tris–HCl pH 7.5, 400 mM NaCl) for 5 min. Slides were then incubated in 0.5% blocking reagents (Roche) in TBS for 1 h followed by a wash in 1% BSA (bovine serum albumin, Sigma-Aldrich), 0.3% (v/v) Triton X-100 (Sigma-Aldrich) in TBS for 30 min. Slides were further incubated in Anti-Digoxigenin-AP Fab fragments (Roche) : 1% BSA, 0.3% (v/v) Triton X-100 in TBS (1:3000) for 90 min, followed by two washes in 1% BSA, 0.3% (v/v) Triton X-100 in TBS for 30 min each. For detection, slides were first washed in detection buffer (100 mM Tris–HCl pH 9.5, 100 mM NaCl, 50 mM MgCl_2_) for 5 min, and then incubated in NBT/BCIP (Roche) detection buffer mixture (200 μl NBT/BCIP per 10 ml detection buffer) in dark for 12 h.

All experiments were performed in accordance with relevant institutional guidelines and regulations.

## Supplementary Information

Supplementary Information 1.

Supplementary Information 2.

Supplementary Information 3.

## Data Availability

The data that support the findings of this study are available within the paper and its supplementary materials published online.
