# Supplementary material for: Single-cell transcriptome profiling of buffelgrass (Cenchrus ciliaris) eggs unveils apomictic parthenogenesis signatures
Source: Sci Rep. 2021 May 10;11:9880. doi: 10.1038/s41598-021-89170-y (PMC8110759; doi:10.1038/s41598-021-89170-y)
Supplement: Supplementary file 1 — Supplementary Information 1. [file 41598_2021_89170_MOESM1_ESM.pdf]

Single-cell transcriptome profiling of buffelgrass (*Cenchrus ciliaris*) eggs unveils apomictic parthenogenesis signatures

Authors

Yuji Ke[1], Maricel Podio[2], Joann Conner[1,2], and Peggy Ozias-Akins[1,2]*

Contact information

[1]Institute of Plant Breeding, Genetics & Genomics, [2]Department of Horticulture, University of Georgia, Tifton, GA 31793, USA

*Correspondence: pozias@uga.edu; Tel.: +1-229-386-3902

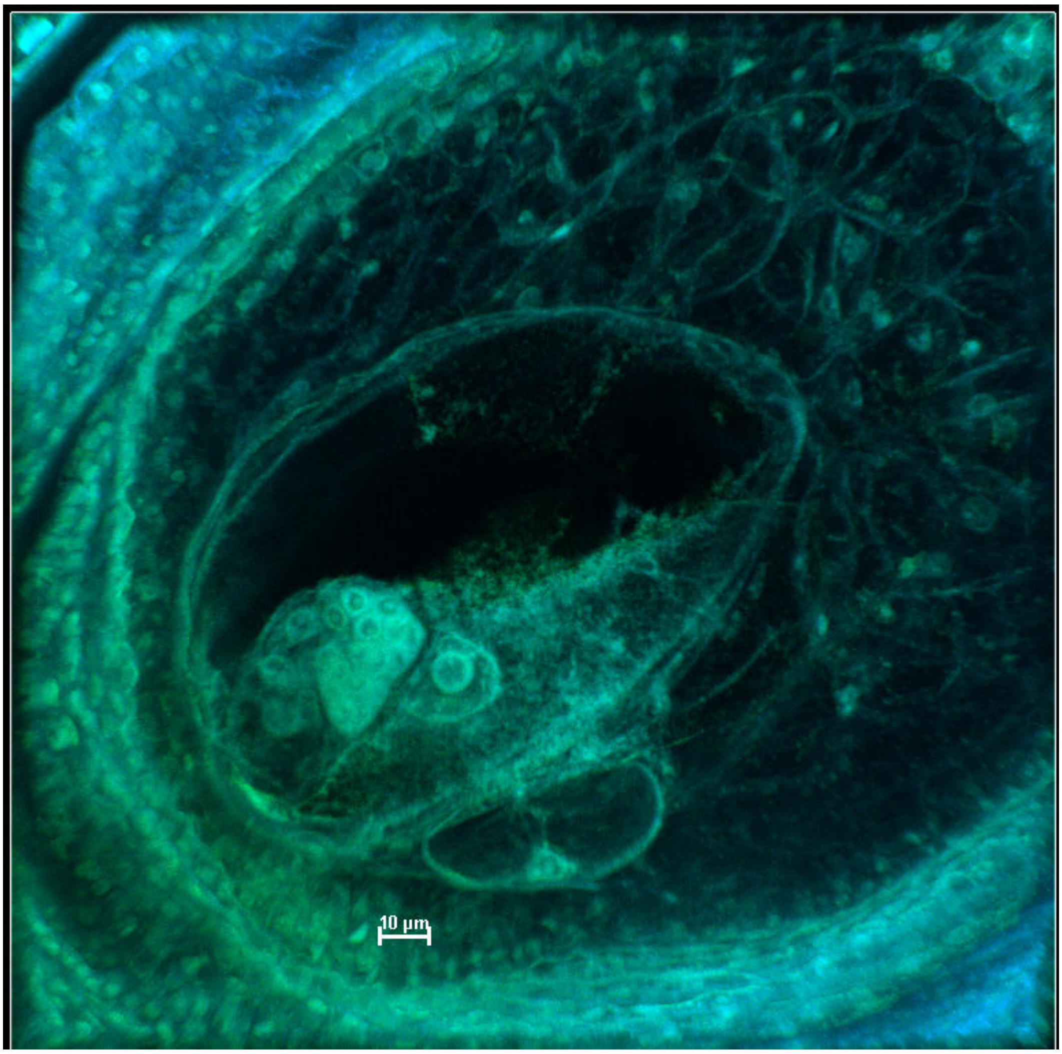

Supplementary Figure 1. Parthenogenetic development of a *C. ciliaris* embryo. Confocal microscopy image from an unpollinated, fixed and cleared apomictic *C. ciliaris* ovary two days after natural anthesis. Polar nucleus is visible beside multicellular proembryo.

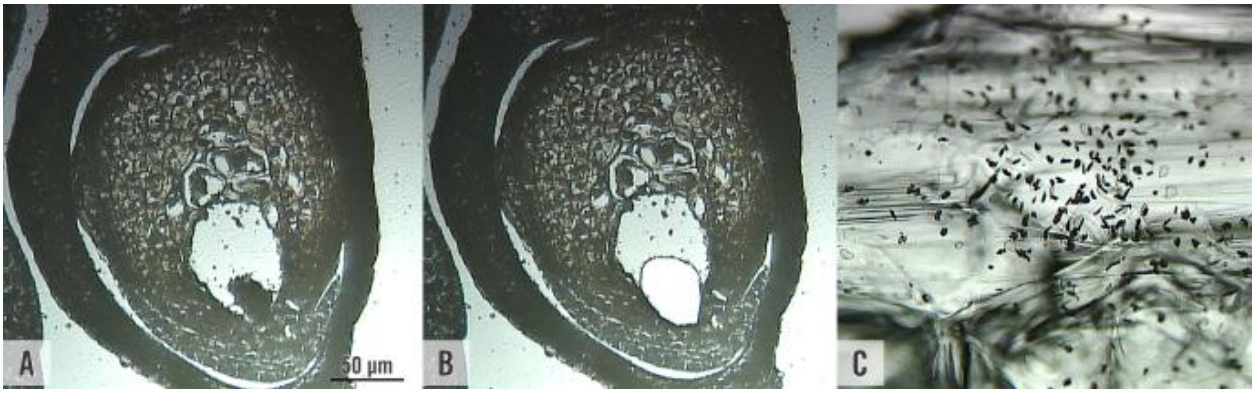

Supplementary Figure 2. Laser capture microdissection under a Leica LMD6000 microscope. (A-B) Sexual ovary section before & after egg cell microdissection. (C) Hundreds of egg cells collected in a PCR tube lid. scale bar = 50 μm.
